# Neuropilin-1 Expression Is Induced on Tolerant Self-Reactive CD8^+^ T Cells but Is Dispensable for the Tolerant Phenotype

**DOI:** 10.1371/journal.pone.0110707

**Published:** 2014-10-24

**Authors:** Stephanie R. Jackson, Melissa Berrien-Elliott, Jinyun Yuan, Eddy C. Hsueh, Ryan M. Teague

**Affiliations:** 1 Saint Louis University School of Medicine, Department of Molecular Microbiology and Immunology, St. Louis, Missouri, United States of America; 2 Saint Louis University School of Medicine, Department of Surgery, St. Louis, Missouri, United States of America; 3 Saint Louis University Cancer Center, St. Louis, Missouri, United States of America; University of Tokyo, Japan

## Abstract

Establishing peripheral CD8^+^ T cell tolerance is vital to avoid immune mediated destruction of healthy self-tissues. However, it also poses a major impediment to tumor immunity since tumors are derived from self-tissue and often induce T cell tolerance and dysfunction. Thus, understanding the mechanisms that regulate T cell tolerance versus immunity has important implications for human health. Signals received from the tissue environment largely dictate whether responding T cells become activated or tolerant. For example, induced expression and subsequent ligation of negative regulatory receptors on the surface of self-reactive CD8^+^ T cells are integral in the induction of tolerance. We utilized a murine model of T cell tolerance to more completely define the molecules involved in this process. We discovered that, in addition to other known regulatory receptors, tolerant self-reactive CD8^+^ T cells distinctly expressed the surface receptor neuropilin-1 (Nrp1). Nrp1 was highly induced in response to self-antigen, but only modestly when the same antigen was encountered under immune conditions, suggesting a possible mechanistic link to T cell tolerance. We also observed a similar Nrp1 expression profile on human tumor infiltrating CD4^+^ and CD8^+^ T cells. Despite high expression on tolerant CD8^+^ T cells, our studies revealed that Nrp1 had no detectable role in the tolerant phenotype. Specifically, Nrp1-deficient T cells displayed the same functional defects as wild-type self-reactive T cells, lacking *in vivo* cytolytic potential, IFNγ production, and antitumor responses. While reporting mostly negative data, our findings have therapeutic implications, as Nrp1 is now being targeted for human cancer therapy in clinical trials, but the precise molecular pathways and immune cells being engaged during treatment remain incompletely defined.

## Introduction

Activated cytotoxic T cells represent a powerful branch of the adaptive immune system, capable of detecting cellular abnormality and protecting the human host from microbial threats and malignancy. These cells are armed with a plethora of effector mechanisms, including cytolytic molecules and proinflammatory cytokines. While critical for host defense, CD8^+^ T cell responses can be detrimental or even fatal when deregulated [1], [2], [3], [4], [5]. Thus, T cell activation following antigen engagement must be tightly controlled. One mechanism of control is peripheral T cell tolerance, which is critical in preventing immunopathology mediated by excessive CD8^+^ T cell activity, and is especially important to limit the activation of self-reactive T cells harbored in the periphery of healthy individuals [6]. However, tolerance also presents a formidable barrier to eliciting anti-tumor immune responses since many cancer antigens are also expressed in healthy self-tissue [7]. In an effort to improve treatment options for patients with cancer, extensive work has gone into characterizing the factors that lead to T cell tolerance and the development of strategies that break tolerance toward tumor/self-antigens to augment immunotherapy [8].

We and others have reported that CD8^+^ T cell tolerance is regulated in part by the coinhibitory surface receptors PD-1, LAG-3 and CTLA-4 [9], [10], [11]. In our studies, these proteins were upregulated after antigen priming, particularly under tolerant conditions where these molecules proved fundamental for the dysfunctional phenotype [9]. Although not characterized as a coinhibitory receptor, a similar pattern of expression was also observed for the surface molecule, neuropilin-1 (Nrp1), implying a possible link between Nrp1 and the induction or maintenance of CD8^+^ T cell tolerance.

Nrp1 is a type-I transmembrane glycoprotein originally discovered for its role in neuron axon guidance and embryonic vessel formation [12], [13], [14], [15]. Its expression has subsequently been reported on malignant cells and several immune cell subsets including dendritic cells (DC), conventional T cells, and regulatory T cells (Treg) [16], [17], [18]. Nrp1 has three extracellular domains important for ligand binding and receptor dimerization, and a short cytoplasmic tail that lacks a kinase domain. To support downstream signaling, Nrp1 dimerizes with other surface proteins such as plexin molecules, VEGFR2, TGFB-R, EGFR, HGFR, and PDGFR-α, allowing strong interactions with the multiple ligands. These varied binding partners permit Nrp1 ligation to modulate a variety of signaling pathways, contributing to the remarkable diversity in the physiological activities attributed to Nrp1 [16].

The first description of a possible role for Nrp1 in the immune system showed homotypic interactions between Nrp1 on mature DC and human T cells in the initiation of the primary T cell immune response [18]. Subsequent studies focused primarily on Nrp1 in murine Treg, as antigen engagement selectively supports Nrp1 expression on Treg versus conventional CD4^+^ T cells [17]. In Treg, Nrp1 promotes synapse formation with DC and longer, more stable interactions leading to enhanced suppression [19]. The first *in vivo* reports found that Nrp1 helped suppress autoreactive CD4^+^ T cells in a murine experimental autoimmune encephalomyelitis model [20]. More recently, Nrp1 was identified as a marker of natural versus induced Treg and shown to play a role in contact-independent suppression mediated by these cells [21], [22]. Mechanistic studies by Delgoffe and colleagues demonstrated a role for Nrp1 in the stability and suppressive activity of Treg, involving phosphatase PTEN recruitment to the immunological synapse via association with the PDZ- protein interaction domain encoded in the cytoplasmic tail of Nrp1 expression [23]. Thus, there is a building consensus that Nrp1 is important for activation, synapse formation, and suppressive activity of CD4^+^ Treg.

Few reports have explored the biology of Nrp1 on CD8^+^ T cells. In a thorough analysis of the genes involved in CD8^+^ T cells memory formation, Kaech *et al*. reported modest Nrp1 upregulation (2-fold higher) on effector and memory CD8^+^ T cells relative to naive cells [24]. In 2012, *Hansen et al* comment briefly that a minor population of CD8^+^ T cells in the spleen expressed Nrp1 (1%), which contrasted with 80% of Foxp3^+^ Treg and 23% of CD4^+^ T cells [25]. Nrp1 has been suggested as a potential marker for liver-sinusoidal endothelium-primed CD8^+^ T cells that escape deletional tolerance [26]. These Nrp1^+^ T cells formed a distinct memory cell population that lacked cytokine responsiveness, representing the first correlation between Nrp1 expression and CD8^+^ T cells with a dysfunctional phenotype. However, these reports were correlative, and the functional relevance of Nrp1 in regard to CD8^+^ T cell immunity and tolerance has not yet been determined.

In this study, we investigated the expression profile and functional role of Nrp1 on CD8^+^ T cells under naive steady state conditions, and within distinct *in vivo* environments where antigen was encountered within immune, inflammatory, or tolerant contexts. We report that Nrp1 was selectively induced and highly expressed on CD8^+^ T cells engaging self-antigen, both in mice and in human melanoma infiltrating T cells. Despite this unique expression pattern, Nrp1 appeared to play no part in the dysfunctional phenotype of murine self-reactive T cells. This was confirmed in Nrp1-deficient T cells, which performed similarly to wild-type T cells within both immune and tolerant environments, and when used in adoptive immunotherapy for cancer. These results support Nrp1 as a potential biomarker for dysfunctional self-reactive CD8^+^ T cells, which is dispensable for the induction and maintenance of T cell tolerance.

## Results

### Nrp1 is highly expressed on tolerant CD8^+^ T cells primed by self-antigen in healthy hepatocytes

**Figure 1:**
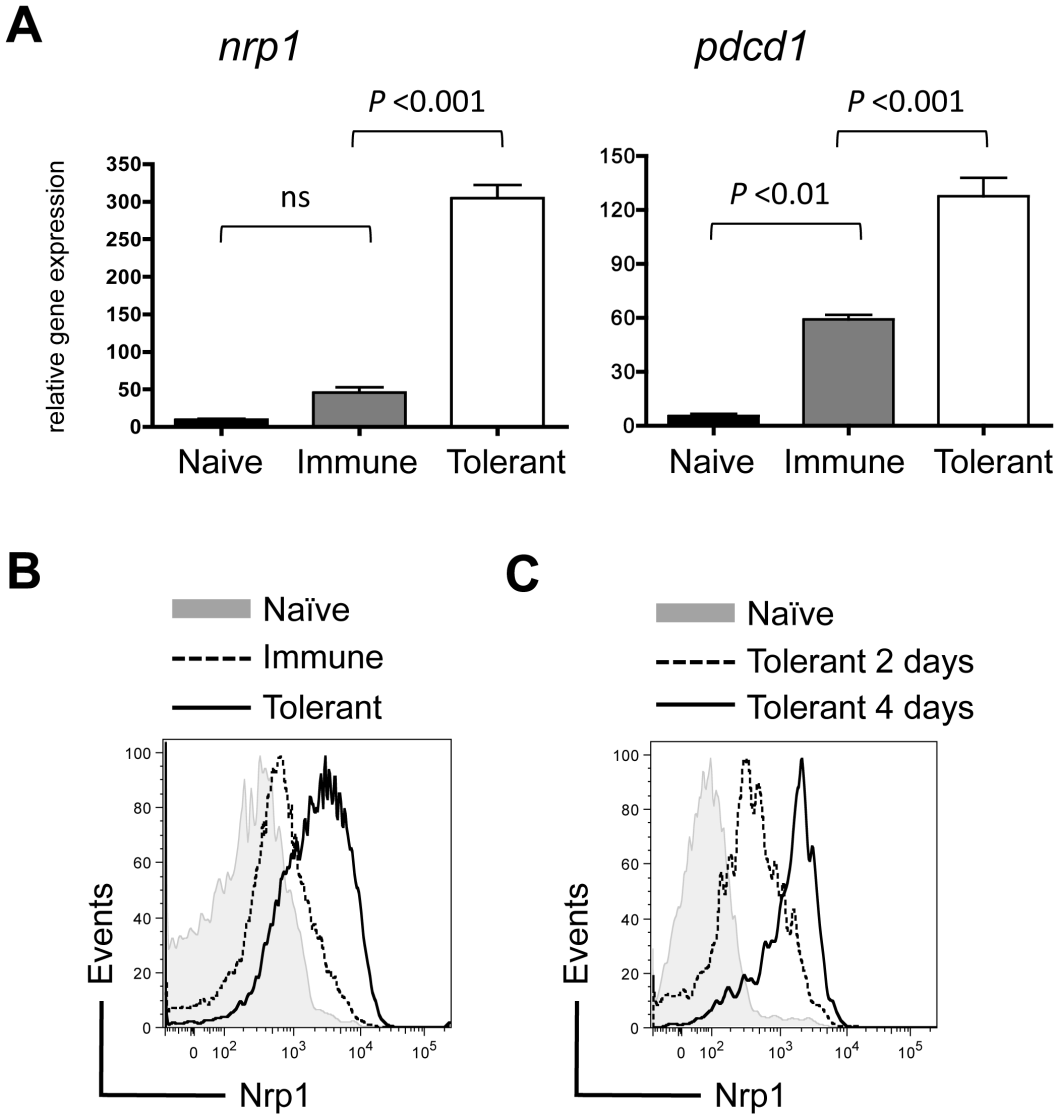
Nrp1 is highly expressed on tolerant CD8^+^ T cells primed by a self-antigen in healthy liver. Naive Gag-specific CD8^+^ T cells (CD90.1^+^) were transferred into B6 mice (naive), B6 mice bearing an immunogenic FBL tumor (immune), Alb∶Gag mice (tolerant). (A) Two days after transfer, T cells were purified by cell sorting and RNA isolated for gene expression by microarray. Graphs displays *nrp1* and *pdcd1* relative gene expression pooled from biological triplicate samples, and error bars represent SD. (B) Nrp1 protein expression on T cells 3 days after adoptive transfer into the naive, immune or tolerant environment. Data are representative of 3 experiments, each with 3 recipient mice per group. (C) Nrp1 protein upregulation on T cells 2 and 4 days after transfer into Alb∶Gag recipients. Data are representative of 2 time course experiments.

To define the intrinsic pathways regulating CD8^+^ T cell tolerance versus immunity, the gene expression profiles of Gag-specific CD8^+^ T cells (TCR^Gag^) were defined after transfer into normal B6 mice (naive), B6 mice with an immunogenic Gag-positive FBL tumor (immune), or Alb∶Gag mice that express the same Gag antigen under control of the Albumin promoter in healthy hepatocytes (tolerant) [27]. We previously reported that recognition of Gag in the immune context leads to CD8^+^ T cell expansion, acquisition of effector function, and memory formation. However, in the tolerant context of an Alb∶Gag host, these same T cells proliferate briefly but fail to acquire effector function and are largely deleted 8 days after transfer [9],[28]. These tolerant T cells were characterized by high expression of multiple inhibitory receptors (e.g. CTLA-4, PD-1, LAG-3) vital for their dysfunctional phenotype [9]. Subsequent gene array analysis revealed that expression of the gene that encodes Nrp1 mirrored that of these co-inhibitory receptors on tolerant T cells. Specifically, like PD-1 (encoded by *pdcd1*), *nrp1* gene expression was elevated upon engagement of antigen, but significantly upregulated in T cells engaging antigen under tolerant conditions (Fig. 1A). This expression profile was reflected on the surface of tolerant T cells relative to immunized T cells (Fig. 1B), which was evident as early as 2 days after T cell transfer into the tolerant environment (Fig. 1C). Collectively, these data demonstrate that Nrp1 marks tolerant T cells primed by a self-antigen *in vivo*, and compel further analysis of its possible role in the biology of tolerant CD8^+^ T cells.

### Nrp1 expression promotes optimal CD8^+^ T cell proliferation in response to self-antigen

One of the earliest reported roles for Nrp1 in the immune system is the regulation of T cell proliferation. In these first reports, Nrp1 expression on human dendritic cells and CD4^+^ T cells promoted prolonged T cell∶DC interactions and more extensive proliferation of resting T cells [18]. However, these and other studies were limited only to *in vitro* analysis [19]. In the murine EAE model of multiple sclerosis, the exact opposite trend was observed, with Nrp1-deficient CD4^+^ T cells proliferating more than Nrp1^+^ cells [20]. Thus, there is still uncertainty surrounding the role of Nrp1 in T cell proliferation. More importantly, the impact of Nrp1 expression on CD8^+^ T cell proliferation has not been analyzed. In our model, tolerance induction following engagement of antigen in the liver commences with extensive proliferation of responding CD8^+^ T cells [28]. To examine the contribution of Nrp1 here, we evaluated the proliferation of transgenic Gag-specific T cells deficient for the gene that encodes Nrp1 via Lck-cre mediated gene deletion (Nrp1^f/f^ Lck-cre TCR^Gag^), referred to hereafter as *nrp1^f/f^*.

**Figure 2:**
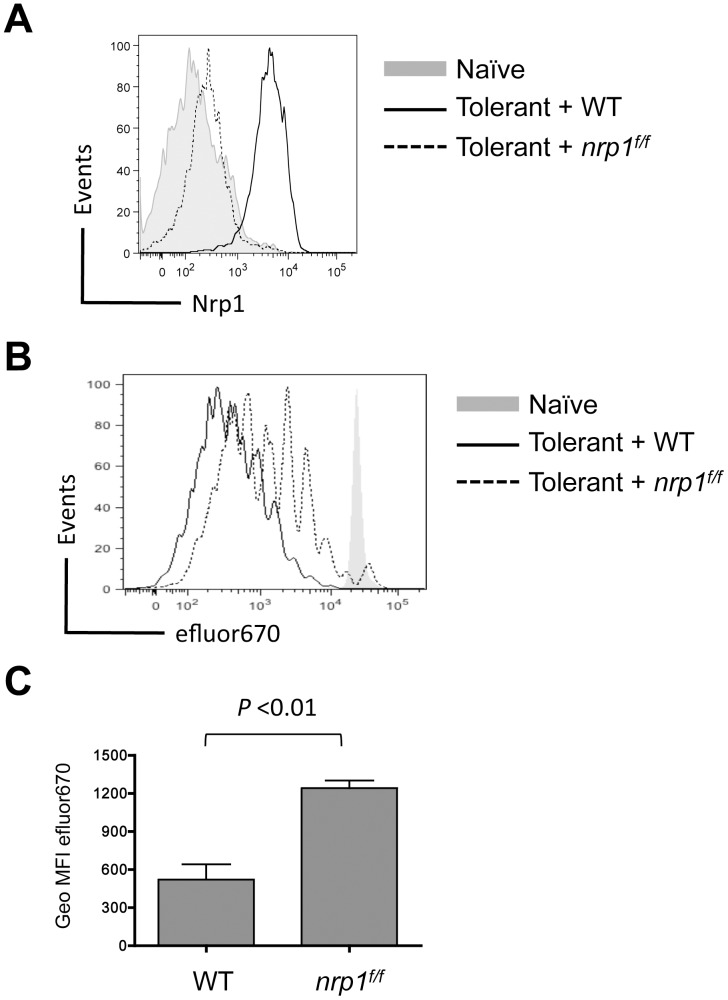
Nrp1 expression corresponds with a modest increase in CD8^+^ T cell proliferation in response to self-antigen. Naive WT or Nrp1-deficient CD8^+^ T cells were labeled with efluor670 cytoplasmic dye and co-transferred into B6 (naive) or Alb∶Gag (tolerant) mice. (A) Nrp1 expression on WT and *nrp1^f/f^* T cells 3 days after transfer is compared in the overlaid histograms. (B) Dilution of efluor670 dye in transferred T cells was assessed 3 days after transfer and is displayed in overlayed histograms. (C) Geometric mean fluorescent intensity of efluor670 in T cells from either WT or Nrp1-deficient T cells (lower dye expression corresponds to more proliferation) is pooled from 4 separate experiments, each with 3 mice per group. Error bars are standard error of the mean (SEM) with *P* value indicated.

WT Gag-specific CD8^+^ T cells (CD90.1^+^) were co-transferred with *nrp1^f/f^* Gag-specific CD8^+^ T cells (CD90.1^+^/90.2^+^) into normal B6 recipients or Alb∶Gag recipients. After 4 days in Alb∶Gag hosts, surface expression of Nrp1 was evident on WT cells, but expression on *nrp1^f/f^* T cells was similar to naive T cells (Fig. 2A). This same *nrp1* expression pattern was reflected at the gene expression level in identically treated cells (Fig. S1). While it is unlikely that Nrp1 expression was completely absent in all *nrp1^f/f^* T cells, we consistently observed a 5-10 fold reduction in surface protein expression relative to WT T cells (Fig. S1). Both sets of transferred T cells underwent several rounds of cell division regardless of genotype but fewer *nrp1^f/f^* T cells went into cell cycle and those that did underwent fewer rounds of division relative to WT T cells (Fig. 2B and 2C). These data demonstrate that Nrp1 is not essential for CD8^+^ T cell proliferation, but may be required for optimal responses, contributing modestly to the extent of CD8^+^ T cell activation during early induction of peripheral self-tolerance.

### Nrp1 does not contribute to deficiencies in effector function by tolerant T cells

**Figure 3:**
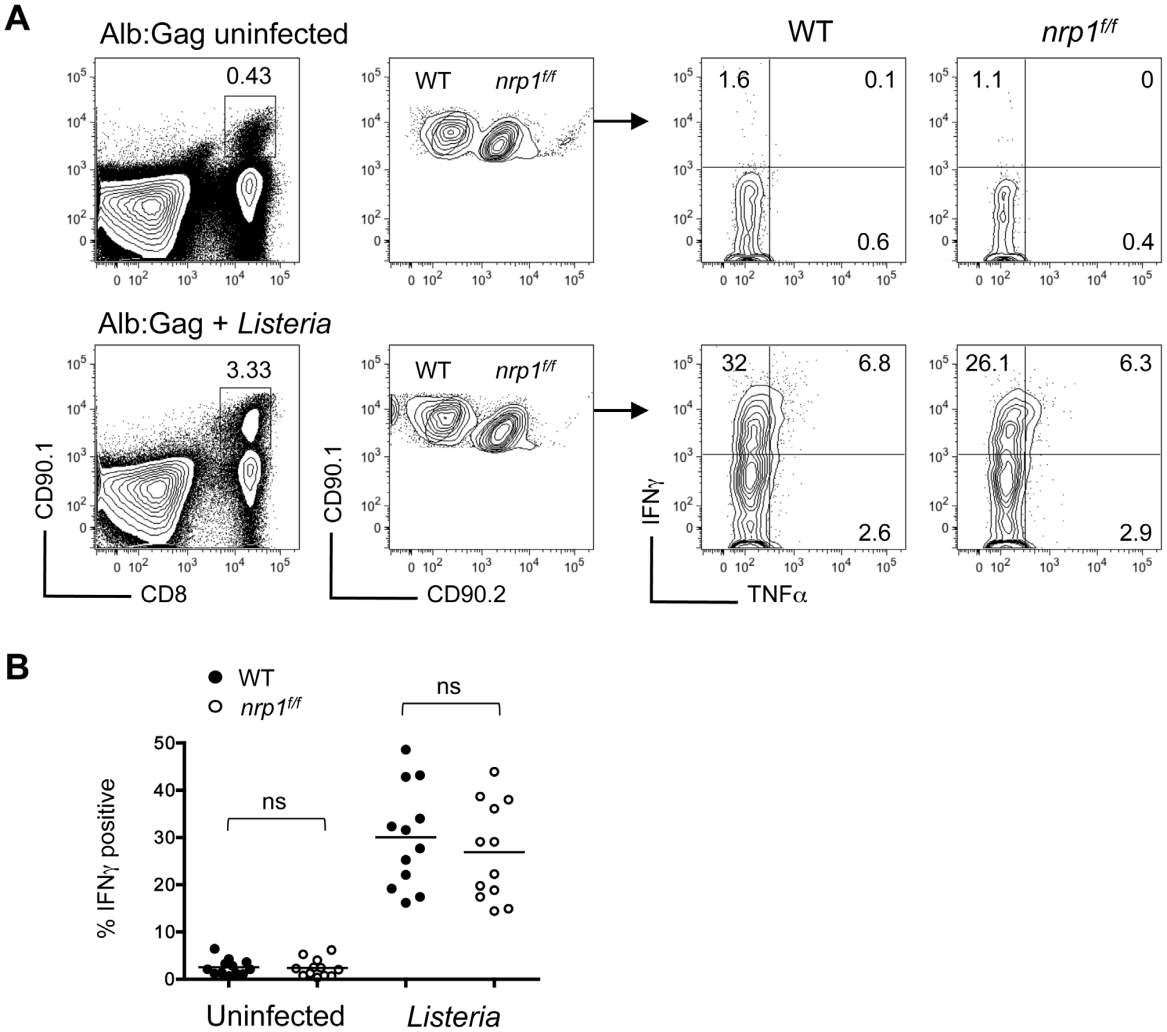
Nrp1 expression does not contribute to the lack of effector function in tolerant CD8^+^ T cells. Naive WT (CD90.1^+^) and Nrp1-deficient (CD90.1^+^/90.2^+^) Gag-specific CD8^+^ T cells were transferred into Alb∶Gag recipients with (lower) or without (upper) a *Listeria* infection. Three days later, production of IFNγ and TNFα by transferred T cells was measured after overnight restimulation with Gag peptide. (A) Plots display T cell frequency (left) and IFNγ and TNFα production (right). Inset numbers are the percent of total splenocytes within the inscribed square region (left). Numbers in each quadrant represent the percent of gated CD8^+^ CD90.1^+^ T cells (right). (B) The percent of gated CD8^+^ CD90.1^+^ or CD8^+^ CD90.1^+^/90.2^+^ T cells that express IFNγ in differentially treated Alb∶Gag recipients was graphed, with each circle representing individual mice pooled from 4 separate experiments. Horizontal bars represent the average for each group (ns  =  not significant).

Negative regulatory receptor signaling has been shown to trigger dysfunction of tolerant CD8^+^ T cells [9]. If Nrp1 expression also contributes to poor effector activity, Nrp1-deficient T cells should have improved cytokine responses following self-antigen encounter. In support of this, studies by Bottcher *et al*. identified a distinct population of Nrp1^+^ CD8^+^ T cells lacking cytokine responsiveness that correlated with high levels of Nrp1expression [26]. To evaluate whether Nrp1 expression directly regulated effector function of tolerant CD8^+^ T cells, WT Gag-specific T cells (CD90.1^+^) were co-transferred with *nrp1^f/f^* CD8^+^ T cells (CD90.1^+^/90.2^+^) into Alb∶Gag hosts with or without acute *Listeria monocytogenes* infection. In agreement with our previous results [28], IFNγ production was not elicited in WT T cells within Alb∶Gag recipients, but could be induced in these same hosts when accompanied by inflammation during an acute *Listeria* infection (Fig. 3). However, the same was true for Nrp1-deficient T cells, suggesting Nrp1 was not involved in the regulation of effector cytokine production by tolerant CD8^+^ T cells, nor was Nrp1 required for self-reactive CD8^+^ T cells to acquire effector functions during *Listeria* infection.

**Figure 4:**
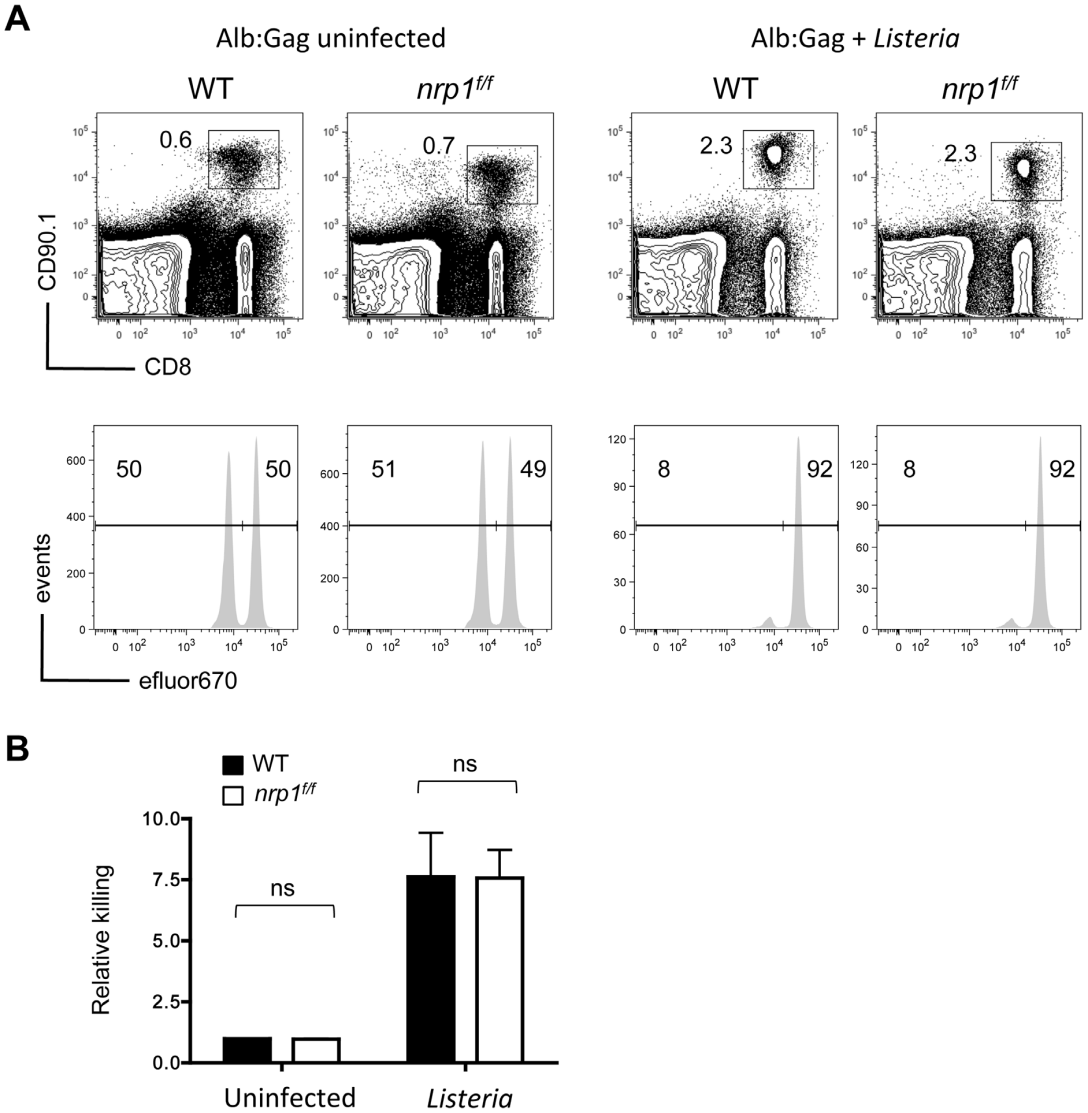
The cytotoxic potential of tolerant CD8^+^ T cells is not regulated by Nrp1. WT or Nrp1-deficient CD90.1^+^ Gag-specific CD8^+^ T cells were transferred into Alb∶Gag recipients with or without *Listeria* infection. Three days after transfer, recipients were infused with a 1∶1 ratio of Gag (eFluor 670^low^) and control (eFluor 670^high^) peptide-pulsed target cells. Twenty hours later (day 4), recipient spleens were harvested and the frequency of transferred Gag-specific T cells was determined by flow cytometry (upper panels). Inset numbers are the percent of total splenocytes within the inscribed regions. Target cell frequency is displayed as histograms with the percentage of total eFluor 670-positive cells inset above the indicated regions (lower panels). (B) Graph displays relative target cell killing (% eFluor 670^high^/% eFluor 670^low^) pooled from 3 independent experiments. Error bars represent standard deviation (ns  =  not significant).

To further characterize the contribution of Nrp1 to the functional defects associated with tolerant self-reactive T cells, *in vivo* cytolytic activity was assessed. WT or *nrp1^f/f^* T cells were transferred separately into Alb∶Gag recipient mice in the presence or absence of *Listeria*, followed 3 days later by an infusion of fluorescently labeled peptide-pulsed target cells. Consistent with our previous data [28], WT Gag-specific T cells failed to kill Gag-pulsed target cells within the tolerant environment, but specific cytolytic activity was induced by self-antigen when encountered in conjunction with *Listeria* (Fig. 4). Cytolytic activity by Nrp1-deficient T cells was essentially identical to WT T cells under these same conditions. Likewise, blockade of Nrp1 by *in vivo* administration of anti-Nrp1 antibodies had no impact on WT T cell effector function (data not shown). These data further support that Nrp1 neither hinders nor facilitates the cytolytic potential of self-reactive CD8^+^ T cells.

### Nrp1 does not influence peripheral deletion of self-reactive CD8^+^ T cells

**Figure 5:**
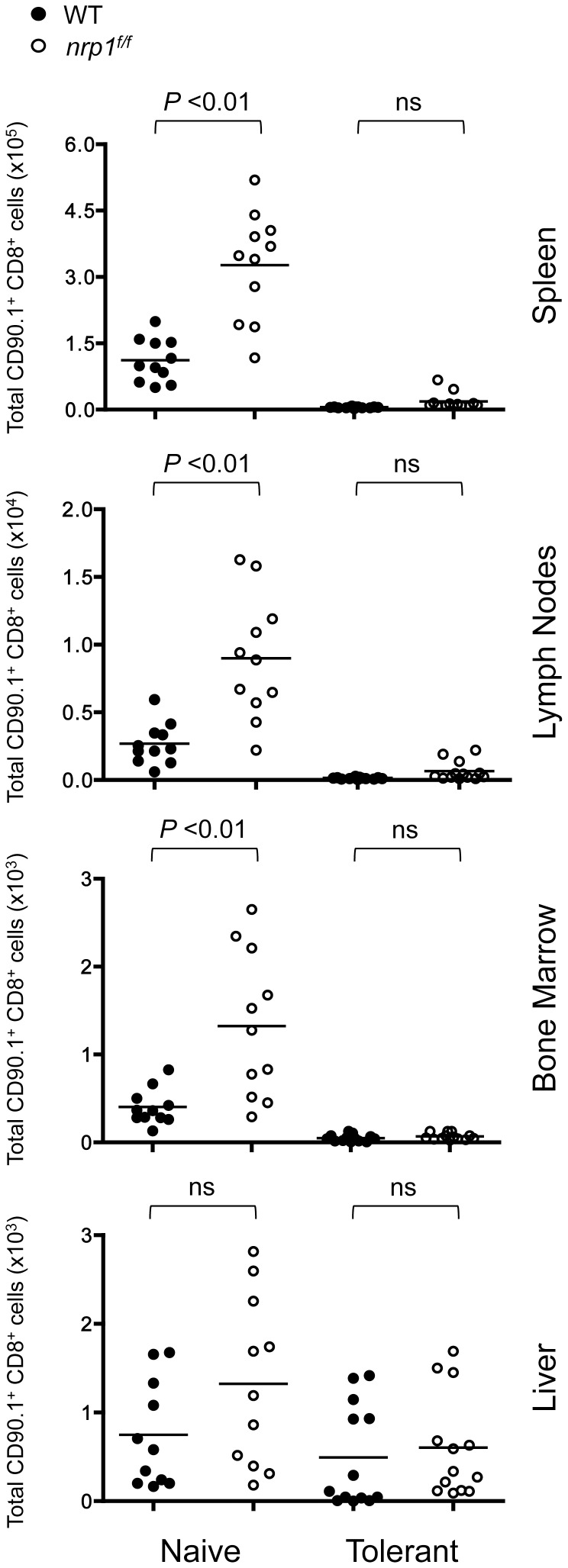
Deletion of self-reactive CD8^+^ T cells is not mediated by expression of Nrp1. Gag-specific CD8^+^ T cells from WT (CD90.1^+^) and Nrp1-deficient (CD90.1^+^/90.2^+^) donor mice were co-transferred into B6 or Alb∶Gag recipients. The frequency of transferred T cells in recipient tissues was assessed after 8 days. The total number of WT or Nrp1-deficinet T cells in the indicated tissue is displayed graphically and shows pooled data from 4 independent experiments, with each circle representing data from one mouse. Horizontal bars represent the average for each group and *P* values are indicated (ns  =  not significant).

In tumor cells, ligation of Nrp1 can either promote cell survival or induce apoptosis, depending on the specific co-receptor and ligand involved [15]. Nrp1 ligation is reported to protect Treg against apoptotic cell death and induce a transcriptional profile enriched in pathways promoting survival and stability [23]. In our model, Nrp1 expressing self-reactive T cells are deleted by Bim-mediated apoptosis approximately 8 days after infusion into Alb∶Gag recipients [9]. To identify any potential role of elevated Nrp1 expression in the elimination of these T cells, WT (CD90.1^+^) and *nrp1^f/f^* (CD90.1^+^/90.2^+^) Gag-specific CD8^+^ T cells were co-transferred into normal B6 or Alb∶Gag host mice. Eight days later, the persistence of these transferred T cells was assessed in spleen, lymph node, bone marrow and liver. Under naive steady-state conditions, *nrp1^f/f^* CD8^+^ T cells persisted slightly better than WT T cells in many of these tissues, but both cell types were deleted within the tolerant environment regardless of genotype (Fig. 5). Similarly, *in vivo* blockade by administration of anti-Nrp1 antibodies was also ineffective at preventing WT T cell deletion (data not shown). It was also evident that Nrp1expression did not affect T cell trafficking, as the relative proportion of WT to *nrp1^f/f^* CD8^+^ T cells remained equivalent in all tissues analyzed. These results suggest that Nrp1 had no role in peripheral deletion of self-reactive CD8^+^ T cells following encounter with self-antigen.

### Ablation of Nrp1 does not improve adoptive T cell immunotherapy for leukemia

**Figure 6:**
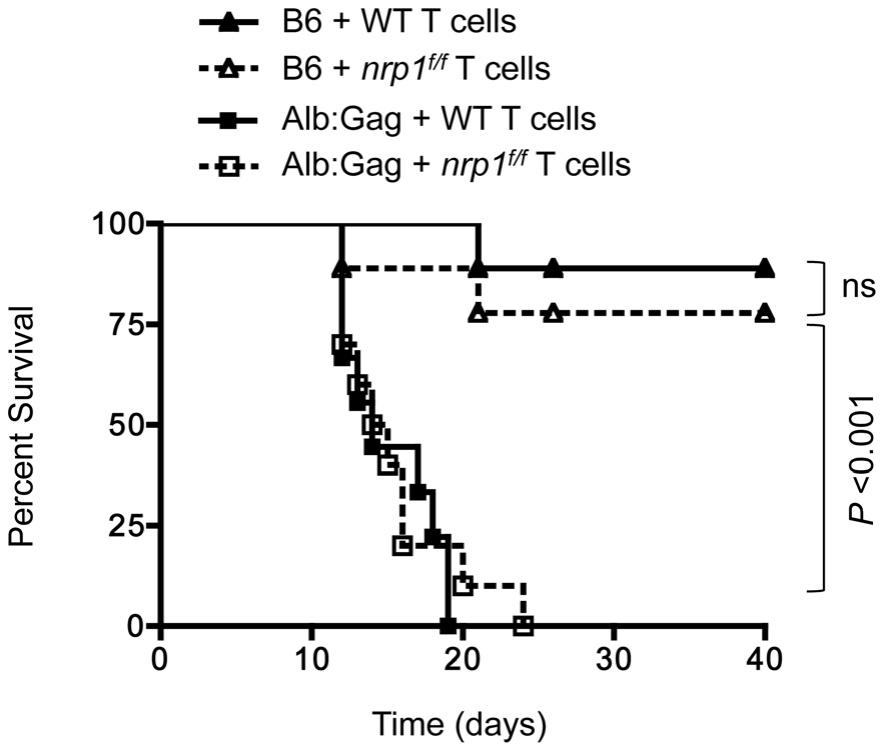
Nrp1 expression on adoptively transferred CD8^+^ T cells does not influence the efficacy of immunotherapy for leukemia. B6 or Alb∶Gag recipient mice were inoculated with FBL leukemia. Seven days later, tumor-bearing recipients were infused with WT or Nrp1-deficient Gag-specific CD8^+^ T cells. Recipient survival was tracked for 40 days, and results pooled from 2 separate experiments are depicted in the graph showing percent survival (*y*-axis) over time in days (*x*-axis), with a total 10 mice in each treatment group. Data from B6 and Alb∶Gag groups were compared and *P* values are indicated (ns  =  not significant).

Together, our data suggest that Nrp1 does not have an important role in the regulation of CD8^+^ T cell function or survival, and has no real influence on the dysfunctional phenotype of tumor/self-reactive CD8^+^ T cells. To definitively assess whether Nrp1 expression impacts CD8^+^ T cell function over time, we directly compared WT and *nrp1^f/f^* T cells for efficacy in our model of adoptive T cell immunotherapy for cancer. A disseminated and progressive murine FBL leukemia was established in normal B6 or Alb∶Gag host mice by i.v. injection, as previously described [28]. One week later, WT or *nrp^f/f^* Gag-specific T cells were infused i.v. into the same recipients, which were monitored for health out to 40 days. Because WT T cells become tolerant in Alb∶Gag recipients [9], [28], adoptive immunotherapy is less effective and these recipients died of progressive tumor in less than 20 days (Fig. 6). Transfer of *nrp1^f/f^* T cells provided a similarly low level of therapeutic benefit to tumor-bearing Alb∶Gag recipients. Conversely, in tumor-bearing B6 recipients, transferred Gag-specific T cells are activated by the immunogenic FBL tumor [9]. Here, such T cells were capable of overcoming established leukemia in a majority (>75%) of recipient mice regardless of Nrp1 expression (Fig. 6). These results reinforce the notion that Nrp1 has no bearing on the *in vivo* persistence, trafficking, or function of CD8^+^ T cells despite high expression on tolerant versus immunized cells (Fig. 1).

### Neuropilin-1 is expressed on human tumor infiltrating T cells

**Figure 7:**
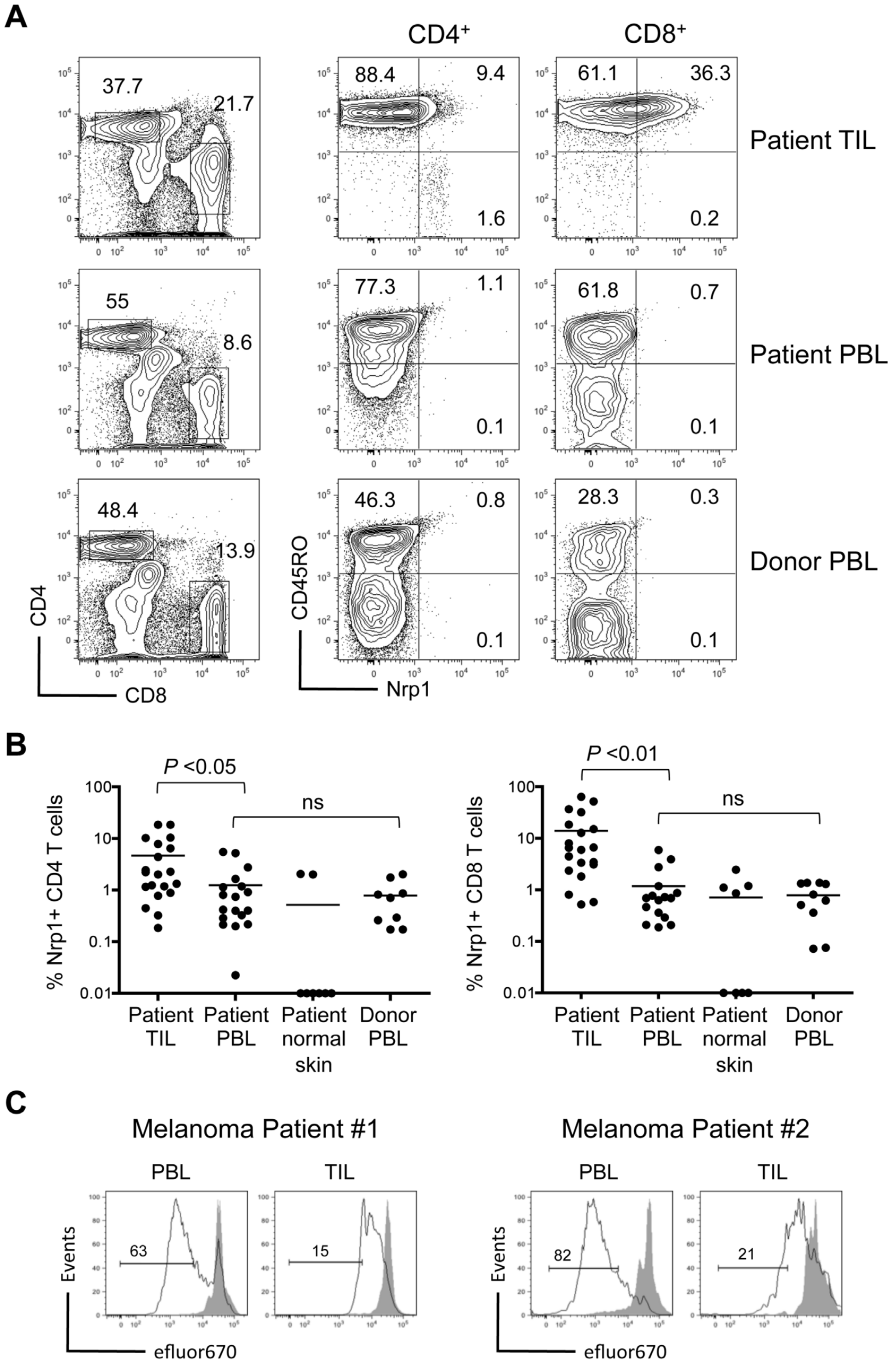
Nrp1 expression is increased on human tumor infiltrating T cells and corresponds with antigen experience. CD45^+^ lymphocytes were analyzed from the blood (PBL) and tumor (TIL) of patients undergoing surgical resection of metastatic melanoma, and compared to lymphocytes from the blood of healthy donors. (A) The frequency of CD4^+^ and CD8^+^ T cells from the indicated tissues was compared (left) and co-expression of CD45R0 and Nrp1 assessed on these gated T cell populations (right). (B) The frequency of Nrp1^+^ T cells among TIL, patient PBL, normal skin tissue adjacent to tumor, or healthy donor PBL is displayed graphically. Results are pooled from 8–20 independent samples, with each circle representing data from one patient/donor. Horizontal bars represent the average for each group and *P* values are indicated (ns  =  not significant). (C) Proliferation of CD8^+^ T cells from patient PBL or TIL was directly compared following 3 day *in vitro* stimulation with anti-CD3/CD28 beads. Histograms show relative dilution of efluor670 dye in stimulated (black line) versus non-stimulated (grey filled peaks) for 2 representative patients. Inset numbers are the percent of cells within the indicated region.

Expression of Nrp1 has been described on a variety of human tumor cells [16], tumor infiltrating Tregs [29], [30], [31], and peripheral blood lymphocytes [16], [18]. However Nrp1 expression on human tumor-infiltrating CD8^+^ T cells has not been reported. Results from our mouse studies suggest that Nrp1 is expressed on tumor/self-reactive T cells, particularly under conditions that favor T cell dysfunction or tolerance. Despite a lack of involvement in the tolerant phenotype, Nrp1 may represent a valuable biomarker for such T cells in cancer patients. We evaluated tumor-infiltrating lymphocytes (TIL) derived from patients undergoing resection of metastatic melanoma. Nrp1 was not detected on the surface of either CD4^+^ or CD8^+^ T cells from the peripheral blood of patients or healthy donors (Fig. 7A). In contrast, Nrp1 was expressed on CD4^+^ and even more so on CD8^+^ TIL in a majority of patient tumor samples (Fig. 7A and 7B). Nrp1 expression on TIL corresponded with an antigen-experienced CD45RO^+^ phenotype (Fig. 7A), implying a possible connection with tumor/self-antigen reactive T cells. Although Nrp1 expression on tolerant mouse T cells did not contribute to the dysfunction, it did provide a unique marker of tumor/self-reactive T cells displaying a tolerant phenotype. To extend this observation to human T cells, *ex vivo* proliferation of CD8^+^ TIL and peripheral blood lymphocytes (PBL) derived from the same patients was compared following stimulation with anti-CD3/CD28 beads. Data from 2 representative patients clearly demonstrates less proliferation from Nrp-1^+^ tumor-infiltrating CD8^+^ T cells relative to Nrp1-negative T cells isolated from PBL (Fig. 7C). Thus, while a case can be made that Nrp1 expression correlates with dysfunctional or tolerant T cells, the precise molecular mechanisms that lead to elevated Nrp1 under these conditions, and how Nrp1 contributes, if at all, in the process remains unknown.

## Discussion

The receptor Nrp1 has been widely studied for its role in regulatory CD4^+^ T cells [17], [19], [23], [32]. However, the precise mechanism by which Nrp1 is involved in immunosuppression remains unclear. Nrp1 is also expressed on other cells of the immune system, but how Nrp1 contributes to the biology in these diverse populations is unknown. In our study of CD8^+^ T cell tolerance, the negative regulatory receptors PD-1, CTLA-4, and LAG-3 were uniquely upregulated on CD8^+^ T cells engaging self-antigen [9]. Similarly, Nrp1 was highly upregulated on the same T cells. Thus, we hypothesized that Nrp1, like these other molecules, might have an important role in the induction and/or maintenance of peripheral CD8^+^ T cell tolerance.

Tolerant Nrp1^+^ T cells have characteristic defects in the ability to produce effector cytokines, perform cytolytic functions, and are typically deleted from the periphery within 8 days of primary self-antigen encounter [9], [28]. Therefore, we expected that a deficiency in Nrp1 might alter the induction of tolerance similar to what has been described when cells engage antigen in the absence of other negative regulatory receptors [33]. Instead, lack of Nrp1 expression had no impact on self-reactive T cells, which still failed to acquire effector capabilities and were deleted by apoptosis identically to WT self-reactive T cells. Additionally, Nrp1 deficiency did not affect the ability to boost self-reactive CD8^+^ T cell activation under inflammatory conditions. Thus, in our model, Nrp1 held no perceivable influence over the phenotype and function of tolerant self-reactive CD8^+^ T cells.

Coinhibitory molecules play an immunoregulatory role in controlling the balance between T cell activation and tolerance. Manipulation of these coinhibitory pathways provides a means to enhance immune responses to promote anti-tumor immunity [34]. Although antitumor immunity is often limited by self-tolerance, the outcome of antigen engagement within tumor versus healthy self-tissue can be quite different due to differences in costimulatory and coinhibitory signals received. The additional inflammatory and immunosuppressive mediators present in a tumor environment might alter the threshold for tumor/self-reactive T cell activation. Conversely, a role for Nrp1 expression on CD8^+^ T cells that is distinct from tolerance might emerge under conditions in which tumor is present. To assess a potential role for Nrp1 in CD8^+^ T cell function under these conditions, we evaluated the ability of Nrp1-deficient T cells to provide effective adoptive T cell immunotherapy for cancer. This is of special interest since anti-Nrp1 antibodies have recently completed phase I (NCT00747734) and phase Ib (NCT00954642) trials for patients with advanced solid tumors [35], [36]. Thus, characterizing the immune subsets that anti-Nrp1 might target is important for evaluating potential mechanism of action and off-target toxicities. In our murine immunotherapy experiments, Nrp1 expression on adoptively transferred T cells had no influence on recipient survival outcomes, with treated mice uniformly succumbing to tumor under tolerant conditions but able to overcome the same tumors under immune conditions regardless of Nrp1 expression. Collectively, these results support that expression of Nrp1 on CD8^+^ T cells does not play a role in the tolerant phenotype that normally limits responses during adoptive T cell immunotherapy for cancer in mice.

In evaluations of Nrp1 on human CD8^+^ T cells, we observed that some melanoma tumor-infiltrating T cells displayed significantly upregulated Nrp1 relative to T cells from normal adjacent skin tissue or peripheral blood. Thus, a correlation between Nrp1 expression and tumor/self-reactive could be made. However, the role Nrp1 plays in the biology of human CD4^+^ and CD8^+^ TIL remains unknown. Additionally, the tumor-derived factors that lead to elevated Nrp1 in some but not all patients with similar cancers have not been defined and warrant further investigation.

While we did not identify a role for Nrp1 in the regulation of CD8^+^ T cell function or survival in a tolerant environment, our results should not be interpreted to imply a lack of utility for Nrp1 blockade in cancer therapy. Nrp1 function is complex, in part due to the multiplicity of co-receptors and ligands that Nrp1 can interact with, and its broad expression on a variety of cell types [17], [18], [37]. The impetus for using Nrp1 antibody blockade in cancer derives from its direct role in tumor cell survival and proliferation [38], [39], its role in VEGF ligand-mediated angiogenesis [15], [40], [41], [42], and also in the augmentation of Treg suppression [16], [19], [32]. Thus, Nrp1 may represent a direct tumor target and also a target for immunotherapy by modulation of Treg activity.

Our study evaluated the role of Nrp1 on tumor/self-reactive CD8^+^ T cells, which has previously been underexplored. We conclude that the expression of Nrp1, although highly elevated on T cells engaging self-antigen in a tolerant context, does not regulate CD8^+^ T cell tolerance. This has important clinical repercussions, as Nrp1 is being targeted therapeutically in cancer patients but the mechanism by which this strategy might enhance anti-tumor immunity is not completely understood. Additionally, while our results suggest Nrp1 may not represent a viable target for manipulation of self-reactive CD8^+^ T cells during cancer immunotherapy, Nrp1 could represent a useful target in other scenarios, such as autoimmunity [20], [43], or as a potential biomarker to help identify autoreactive T cells in patients.

## Materials and Methods

### Mice

*Rag1* ^−/−^ TCR^Gag^ transgenic mice have been previously described [9], [28]. Alb∶Gag mice express the H-2^b^-restricted Friend murine leukemia virus-derived Gag glycoprotein in healthy hepatocytes under control of the Albumin promoter, as previously demonstrated [27]. C57BL/6 (B6) and Lck-cre mice were purchased from The Jackson Laboratory. Nrp1^f/f^ mice were graciously provided by Dr. David Ginty and have previously been described [44]. Nrp1^f/f^ and Lck-cre mice were crossed with *rag1* ^−/−^ TCR^Gag^ transgenic mice. To generate Gag-specific CD8^+^ T cells deficient for neuropilin, Nrp1^f/f^ x TCR^Gag^ mice were crossed with Lck-cre x TCR^Gag^ mice to generate Nrp1^f/f^ x Lck-cre x TCR^Gag^ mice, which are *rag1* sufficient. All animals were maintained under specific pathogen-free conditions and used in accordance with our animal protocol approved by the Animal Care Committee of the Department of Comparative Medicine, Saint Louis University School of Medicine (Saint Louis, MO).

### Cell lines, peptides, and antibodies

FBL is a murine erythroleukemia cell line of B6 origin that expresses the H-2^b^-restricted Friend murine leukemia virus-derived Gag glycoprotein as a tumor associated antigen [9], [28]. Gag peptide (CCLCLTVFL) and ovalbumin (SIINFEKL) control peptides were purchased from Pi Proteomics. Cell culture was conducted in complete RPMI-1640 with 10% FBS (Sigma). Fluorochrome-conjugated antibody to mouse CD8 (53–6.7) was purchased from eBiosciences. Fluorochrome-conjugated mouse antibodies to CD90.1 (OX-7), IFNγ (XMG1.2), TNFα (MP6-XT22), and human antibodies to CD4 (RPA-T4), CD8 (RPA-T8), and CD45RO (UCHL1) were purchased from BD Biosciences. Fluorochrome conjugated antibodies to mouse neuropilin-1 (761705) and human neuropilin-1 (446921) were purchased from R&D Systems. efluor-670 cell dye was purchased from Invitrogen. Anti-mouse Nrp-1A blocking antibody was provided by Genentech and administered intraperitoneally at 10 mg/kg every other day, similar to studies previously described [45].

### T cell transfer, tumor inoculation, infection

Gag-specific T cells were isolated from spleens and lymph nodes of *rag1* ^−/−^ TCR^Gag^ or Nrp1^f/f^ Lck-cre TCR^Gag^ donors. Whole-cell suspensions containing 2×10^6^ Vα3-TCR^+^ CD8^+^ cells were intravenously injected into sex and age (6–8 week) matched recipients. In some experiments, transferred cells were labeled with efluor670 proliferation dye (eBioscience) before infusion according to the manufacturer's protocol. To provide an immunogenic environment, 5×10^6^ FBL cells were established intraperitoneally in B6 recipients 3 days before T cell transfer. Infected recipients received 2×10^6^ CFU attenuated (ΔActA) *Listeria monocytogenes* intravenously immediately prior to T cell transfer.

### Gene array

Naive Gag-specific T cells were sorted or transferred into B6 mice with established FBL tumor (immune) and Alb∶Gag mice (tolerant). Two days after T cell transfer, recipient spleen and lymph node were harvested and pooled. Transferred cells were sorted on a FACSAria (BD Biosciences). RNA was isolated using RNeasy Plus Mini Kit (Qiagen). Total RNA was used to generate aRNA (Affymetrix) and hybridized to the GeneChip Mouse Genome 430 2.0 Array (Affymetrix) and analyzed with a GeneChip scanner 3000 7G (Affymetrix). Data were obtained from 3 biological replicates per condition, and has been deposited in the Gene Expression Omnibus (GEO) with accession code GSE58722.

### Flow cytometry

Recipient spleen and peripheral lymph nodes were harvested for analysis at indicated time points. Tissues were homogenized into single-cell suspensions before cell culture or staining for flow cytometry. Cell suspensions were stained for extracellular markers at 4°C for 30 minutes. *Ex vivo* cytokine production was assessed following overnight stimulation with 4 µg/mL Gag or Ova peptide in the presence of GolgiPlug (BD Biosciences). For intracellular staining of IFNγ and TNFα, cells were fixed and permeabilized in Cytofix/Cytoperm buffer (BD Biosciences), and proteins stained in Perm/Wash buffer (BD Biosciences) for 30 minutes at 4°C according to the manufacturer's protocol. All flow cytometry was conducted using either an LSR II or FACSCanto II (BD Biosciences), and resulting data analyzed using FlowJo software (Tree Star).

### *In vivo* killing assay

Recipient mice received adoptive T-cell transfers and infections as described above. Three days after T cell transfer, B6 splenocytes (targets) were pulsed with 10 µg/ml Gag or control Ova peptide, and differentially labeled with 1 µM or 5 µM eFluor 670, respectively. Targets were then washed twice in phosphate buffered saline, combined at a 1∶1 ratio, and injected into recipient mice intravenously. Twenty hours later, the frequency of eFluor^high^ versus eFluor^low^ targets from recipient spleens was assessed by flow cytometry.

### Immunotherapy assay

One week prior to treatment, FBL leukemia was established in B6 or Alb∶Gag mice by intravenous injection of 0.9×10^5^ viable FBL tumor cells. Seven days after tumor inoculation, tumor-bearing mice received adoptive transfer of 2×10^6^ Gag-reactive CD8^+^ T cells by intravenous injection. Recipient survival was tracked for a minimum of 40 days with daily health monitoring, and recipients were euthanized upon moribund appearance.

### Quantitative RT-PCR

Transferred T cells were sorted to better than 95% purity using a FACSAria III (BD Biosciences). Total RNA was isolated from sorted cells using RNeasy Mini Kit (Qiagen) and cDNA was synthesized using Transcriptor First Strand cDNA synthesis kit (Roche). Real-time PCR was performed with SYBR Select Master Mix (Life Technology) on a 7500 Real-Time PCR System (Applied Biosystems). Relative amplification values were calculated by normalizing to amplification of beta actin. The following primers were used: Nrp1 sense primer: 5′-CACCAACCCCACAGATGTTGT-3′, Nrp1 antisense primer: 5′-CCAACATTCCAGAGCAAGGAT-3′.

### Statistical analysis

The Kruskal–Wallis test was used for statistical comparison (GraphPad Prism 4) of total cell numbers between different treatment groups. A one-way ANOVA was used for statistical comparison of cell frequencies between multiple treatment groups. Survival data were analyzed with a Mantel-Cox log-rank test. *P* values less than 0.05 were considered significant.

### Patients and normal donors

Following a research protocol approved by the Saint Louis University School of Medicine Institutional Review Board (IRB), peripheral blood, tumor, and normal tumor-adjacent tissue were obtained from patients undergoing resections for metastatic melanoma. Blood samples were also obtained from normal healthy volunteers as separate controls. All subjects provided informed written consent by completion of an IRB approved consent form prior to participation in the study.

### Isolation of human TIL and PBMC

Heparinized blood was diluted 1∶1 (v/v) with PBS before Ficoll density centrifugation. The buffy coat containing PBMC was harvested, and washed twice in cold PBS and subjected to cell surface staining and flow cytometry analysis. Fresh pieces of tumor and normal adjacent tissue were minced into 1-mm-size pieces and tissue subjected to mechanical dissociation over 40 µm cell strainer. The resulting cellular suspension was diluted 1∶1 (v/v) with PBS before Ficoll density centrifugation. The buffy coat containing TIL was harvested, washed twice in cold PBS, stained for expression of cell surface markers, and subjected to flow cytometry analysis.

## Supporting Information

Figure S1 **Nrp1 expression is significantly compromised in** ***nrp1^f/f^*** **T cells.** Naive WT or Nrp1-deficient CD8^+^ T cells were transferred into B6 (naive) or Alb∶Gag (tolerant) mice. (A) After 3 days, WT and *nrp1^f/f^* T cells were sorted to better than 95% purity and mRNA isolated for quantitative RT-PCR analysis. Relative *nrp1* gene expression in WT and *nrp1^f/f^* T cells from the indicated environments is shown. Samples were performed in triplicate and error bars represent standard deviation (B) Naive WT or *nrp1^f/f^* CD8^+^ T cells were transferred into B6 (naive) or Alb∶Gag (tolerant) mice. The geometric mean fluorescent intensity of anti-Nrp1-FITC (closed circles) and isotype-Ig (open circles) surface staining is shown for each of 3 separate mice per group. Horizontal bars represent the average for each group and *P* values are indicated (ns  =  not significant).
(TIF)
