# Supplementary figures and images for: Neuropilin-1 Expression Is Induced on Tolerant Self-Reactive CD8+ T Cells but Is Dispensable for the Tolerant Phenotype
Source: PLoS One. 2014 Oct 24;9(10):e110707. doi: 10.1371/journal.pone.0110707 (PMC4208794; doi:10.1371/journal.pone.0110707)

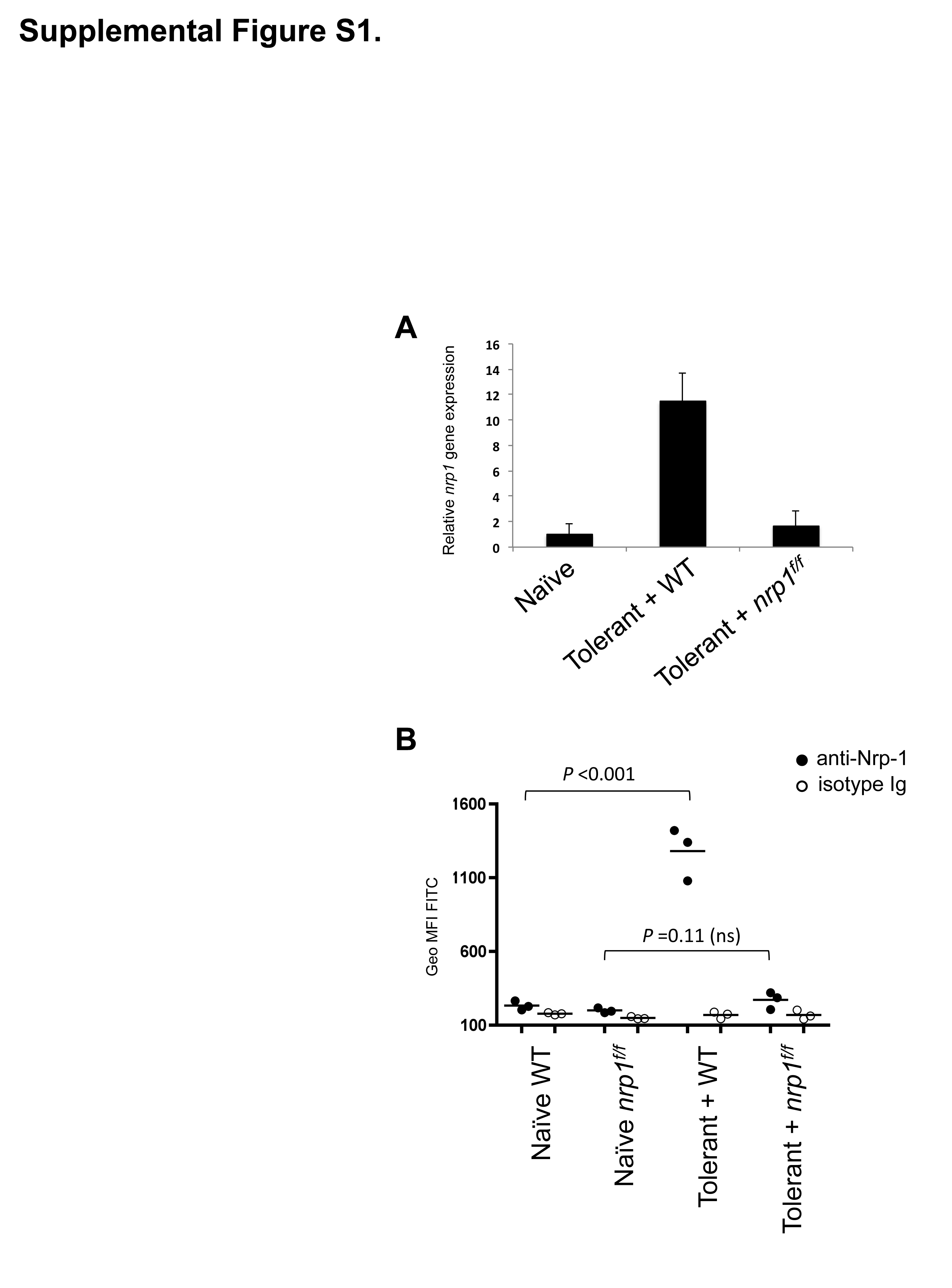

Supplement: Figure S1 — Nrp1 expression is significantly compromised in nrp1f/f T cells. Naive WT or Nrp1-deficient CD8+ T cells were transferred into B6 (naive) or Alb∶Gag (tolerant) mice. (A) After 3 days, WT and nrp1f/f T cells were sorted to better than 95% purity and mRNA isolated for quantitative RT-PCR analysis. Relative nrp1 gene expression in WT and nrp1f/f T cells from the indicated environments is shown. Samples were performed in triplicate and error bars represent standard deviation (B) Naive WT or nrp1f/f CD8+ T cells were transferred into B6 (naive) or Alb∶Gag (tolerant) mice. The geometric mean fluorescent intensity of anti-Nrp1-FITC (closed circles) and isotype-Ig (open circles) surface staining is shown for each of 3 separate mice per group. Horizontal bars represent the average for each group and P values are indicated (ns = not significant). (TIF) [file pone.0110707.s001.tif]
